# Species Tree Estimation for the Late Blight Pathogen, *Phytophthora infestans*, and Close Relatives

**DOI:** 10.1371/journal.pone.0037003

**Published:** 2012-05-17

**Authors:** Jaime E. Blair, Michael D. Coffey, Frank N. Martin

**Affiliations:** 1 Department of Biology, Franklin & Marshall College, Lancaster, Pennsylvania, United States of America; 2 Department of Plant Pathology & Microbiology, University of California Riverside, Riverside, California, United States of America; 3 United States Department of Agriculture – Agriculture Research Services (USDA-ARS), Salinas, California, United States of America; Brigham Young University, United States of America

## Abstract

To better understand the evolutionary history of a group of organisms, an accurate estimate of the species phylogeny must be known. Traditionally, gene trees have served as a proxy for the species tree, although it was acknowledged early on that these trees represented different evolutionary processes. Discordances among gene trees and between the gene trees and the species tree are also expected in closely related species that have rapidly diverged, due to processes such as the incomplete sorting of ancestral polymorphisms. Recently, methods have been developed for the explicit estimation of species trees, using information from multilocus gene trees while accommodating heterogeneity among them. Here we have used three distinct approaches to estimate the species tree for five *Phytophthora* pathogens, including *P. infestans*, the causal agent of late blight disease in potato and tomato. Our concatenation-based “supergene” approach was unable to resolve relationships even with data from both the nuclear and mitochondrial genomes, and from multiple isolates per species. Our multispecies coalescent approach using both Bayesian and maximum likelihood methods was able to estimate a moderately supported species tree showing a close relationship among *P. infestans*, *P. andina*, and *P. ipomoeae*. The topology of the species tree was also identical to the dominant phylogenetic history estimated in our third approach, Bayesian concordance analysis. Our results support previous suggestions that *P. andina* is a hybrid species, with *P. infestans* representing one parental lineage. The other parental lineage is not known, but represents an independent evolutionary lineage more closely related to *P. ipomoeae*. While all five species likely originated in the New World, further study is needed to determine when and under what conditions this hybridization event may have occurred.

## Introduction

The reconstruction of accurate species relationships is a prerequisite for the development of evolutionary hypotheses related to the speciation process. Traditionally, a gene tree estimated from a single locus using standard phylogenetic methods has served as a proxy for the species tree. The emergence of rapid and inexpensive sequencing technologies has allowed researchers to analyze large multilocus, even genomic-scale, datasets for phylogenetic analysis across the tree of life (e.g., [1], [2], [3]). However, concerns have been raised about the accuracy of species relationships inferred from concatenation-based analyses even in the face of highly supported multilocus gene trees ([4], [5], see also [6], [7]). Because concatenation-based approaches assume a single underlying topology across all loci, they are unable to accommodate natural gene tree heterogeneity arising from processes such as incomplete lineage sorting (deep coalescence sensu [8]), horizontal gene transfer, hybridization, or introgression. Over the past several years, new methods have been proposed explicitly for the estimation of species trees. Most utilize a multispecies coalescent approach to accommodate incomplete lineage sorting as the major source of discordance among gene trees (reviewed in [9]), and both maximum likelihood [10], [11] and Bayesian [12], [13] frameworks have been developed. All of these methods allow for the decoupling of gene tree reconstruction from species tree estimation, and several incorporate gene tree uncertainty into the species tree estimate [12], [13], [14]. Similarly, the Bayesian concordance approach estimates the dominant phylogenetic history within a set of heterogeneous gene trees, with no assumptions on the reasons for discordance among loci [15], [16]. All methods assume no recombination within loci and free recombination among loci.

The goal of this study was to address the evolutionary history of *Phytophthora infestans* and its closest relatives. As the causal agent of late blight disease in solanaceous plants, including potato and tomato, *P. infestans* is a pathogen of considerable importance both historically and in modern times. The introduction of *P. infestans* into Europe in the mid 1800's caused wide-spread losses of potato crops, exacerbating societal issues of chronic poverty [17]. Even as recently as 2009, the dissemination of infected tomato plants sold by large retail outlets led to significant losses among home gardeners and commercial farms along the US East Coast [18]. Late blight disease can be acute, occurring sporadically depending on location, but with devastating impacts on yield when outbreaks occur [19]. Other locations, such as the central highlands of Mexico, experience late blight as a chronic disease, with consistent annual losses of susceptible cultivars [20]. The disease spreads rapidly under cool, humid conditions when sporangia are produced on infected leaves and splashed or wind-blown onto neighboring plants [21]. Worldwide losses attributed to late blight have been estimated in the $US billions each year, creating a food security issue in developing countries with a higher dependence on potato for subsistence [22].

*Phytophthora infestans* is heterothallic (requires two mating types for sexual reproduction), but world-wide populations are typically clonal; sexually reproducing populations are known from the central highlands of Mexico and from isolated regions in Europe [23]. Previous analyses have shown that *P. infestans* is closely related to four other foliar blight pathogens, which group together as a subclade (C) within *Phytophthora* Clade 1 [24], [25], [26]. Two of these species are found exclusively on central Mexican hosts; *P. mirabilis*, a heterothallic species infecting the four o'clock, *Mirabilis jalapa* [27], and *P. ipomoeae*, a homothallic species found on the sweet potato relative, *Ipomoea longipedunculata* [28]. *Phytophthora phaseoli* is a homothallic species with a world-wide distribution which causes downy mildew disease on *Phaseolus* lima beans [21]. Recently, a new heterothallic species within Clade 1C, *P. andina*, has been formally described from solanaceous hosts in Ecuador, including wild *Solanum* species in the Anarrhichomenum section, tree tomato (*S. betaceum*) and pear melon (*S. muricatum*) [29]. Initially considered *P. infestans* due to similar morphology, early studies of mating type, isozymes, RFLP fingerprints, and mitochondrial haplotypes identified these isolates as genetically distinct [30], [31]; they also did not appear virulent on potato or tomato [29], [31]. Limited molecular evidence from a small number of nuclear loci has suggested that *P. andina* may have arisen from a hybridization event between *P. infestans* and another Clade 1C species [32], possibly *P. mirabilis* [29], [33]. However, previous studies have been unable to resolve the relationships among the Clade 1C species [24], [25], [26], [32], [33], and some authors have questioned the validity of designating *P. andina* as a separate species from *P. infestans* ([34] but see [35]).

Here we have used three distinct approaches to estimate the species tree for *Phytophthora* Clade 1C. While genomic resources have recently become available for four out of the five members [36], [37], we have assembled a modest but carefully curated dataset of both nuclear and mitochondrial loci from multiple isolates per species. Given its likely hybrid origin [32], we have also analyzed separately the two main haplotypes within our *P. andina* isolates to identify the potential parental lineages. Our concatenation-based phylogenetic analyses of both nuclear and mitochondrial loci were unable to resolve species relationships. Multispecies coalescent approaches and Bayesian concordance analysis yielded consistent and moderately supported species trees showing a close association between *P. infestans*, *P. andina*, and *P. ipomoeae*. Our results are consistent with a previous study [32] suggesting *P. andina* has emerged recently as a hybrid between *P. infestans* and an unknown lineage; our species tree analyses indicate that this unknown lineage is more closely related to the homothallic *P. ipomoeae* than to other members of Clade 1C.

## Results

### Marker Selection

A previous study [24] identified 229 potentially informative loci from the nuclear genome sequences of *Phytophthora infestans*, *P. sojae*, and *P. ramorum*. These loci were screened for protein-coding genes containing predicted introns, and six were chosen for this study based on the consistency of PCR amplification and the observed levels of sequence variation. Sequences were also generated for the ribosomal RNA ITS1 and ITS2 regions, the *Piypt1* locus [38], and six nuclear loci previously used in a comprehensive phylogenetic analysis of the *Phytophthora* genus [24]. In addition, four protein coding loci and two non-coding spacer regions from the mitochondrial genome were chosen for analysis based on observed levels of variation in other *Phytophthora* species [39]. A total of 1175 sequences were analyzed for fifteen nuclear and six mitochondrial loci (Table 1; see Table S1 for a complete list of NCBI accession numbers).

**Table 1:** Molecular loci, primers, and amplification conditions used in this study.

	Transcript/Genomic Location^a^				
Locus		Primer Name	Primer Sequence (5′ - 3′)	T_a_	Ref.
*Nuclear*					
**LSU**	52:695870–	LROR-O	ACCCGCTGAACTYAAGC	53	[96]
(28S Ribosomal DNA)	699597	LSU_Fint^c^	CKTTGACGAAATGGAGCGAT		[24]
		LSU_Rint^c^	TTTCCACACCCTAACACTTGC		[24]
		LR6-O	CGCCAGACGAGCTTACC		[97]
**60SL10**	PITG_19121	60SL10_For	GCTAAGTGTTACCGTTTCCAG	53	[24]
(60S Ribosomal protein L10)		60SL10_Rev	ACTTCTTGGAGCCCAGCAC		[24]
**ARP2/3**	PITG_01846	ARP23_For	TAYCCGCCCTACAAGACG	56^d^	this study
(Actin-related protein 2/3 complex)		ARP23_Rev	CTTCTGGGTCTTGGACTGGT		this study
**Beta-tubulin**	PITG_00156	Btub_F1	GCCAAGTTCTGGGAGGTCATC	60	[24]
		Btub_F2^c^	CGGTAACAACTGGGCCAAGG		[26]
		Btub_R2^c^	GATCCACTCAACGAAGTACG		[26]
		Btub_R1	CCTGGTACTGCTGGTACTCAG		[24]
**PUA**	PITG_17779	PUA_For	AGGTCAAGTCCTCGCAGCAG	67	this study
(Conserved Hypothetical Protein)		PUA_Rev	AGGTCGTCRCCMAGGAAGTG		this study
**Enolase**	PITG_03700	Enl_For	CTTTGACTCGCGTGGCAAC	60	[24]
		Enl_Rev	CCTCCTCAATACGMAGAAGC		[24]
**HSP90**	PITG_06415	HSP90_F1	GCTGGACACGGACAAGAACC	62	[24]
(Heat shock protein 90)		HSP90_F1int^c^	CAAGGTGATCCCGGACAAGGC		[24]
		HSP90_F3^c^	ACGCCTCGTTCTACAAGTCG		[24]
		HSP90_F2^c^	ATGGACAACTGCGAGGAGC		[24]
		HSP90_R1^c^	ACACCCTTGACRAACGACAG		[24]
		HSP90_R2	CGTGTCGTACAGCAGCCAGA		[24]
**HGD**	PITG_01851	HGD68_For	TACAAYCGYCACTTCRTCCT	67	this study
(Homogentisate 1,2-dioxygenase)		HGD68_Rev	RCCCTTYTTRGCGTCRTAG		this study
**ITS**	52:79955–	ITS-1	TCCGTAGGTGAACCTGCGG	53	[98]
(ITS1, 5.8S ribosomal RNA, ITS2)	80840	ITS-4	TCCTCCGCTTATTGATATGC		[98]
**TRP1**	PITG_05318	Trp_For	GCCGCCAAGCAGGTCRT	60	this study
(N-(5′-phosphoribosyl)anthranilate isomerase indole-3-glycerol-phosphate synthase)		Trp_Rev	RAYGCTGTTCACCTCSACCA		this study
**P4P5K**	PITG_10980	P4P5K_FL	CTGCTCATYACGGAGCTGAC	67	this study
(Phosphatidylinositol-4-phosphate-5-kinase)		P4P5K_For^c^	GACGGGYAAYCTYTGGAAC		this study
		P4P5K_Rev	TAGTACAGCACCTCGCAACGC		this study
**Pelota**	PITG_04718	Pelota_For	CAAGAAGCAGATCARCGAG	60	this study
		Pelota_Rev	GCTTGAAGTCAATGTGCTG		this study
**Ras**	PITG_03392	Ras_For	CGTGTCTGCTTCTCCGTTTCG	55	[70]
(Rab1 family GTPase PiYPT1)		Ras_Rev	CCAGGCTTTCGGCAAATTCC		[70]
**Ras Intron**	4:1581350–	RasInt_For	TTGCAGCACAACCCAAGACG	55	[70]
(Rab1 family GTPase PiYPT1, intron 1)	1581696	RasInt_Rev	TGCACGTACTATTCGGGGTTC		[70]
**TigA**	X64537.1^b^	Tig_For	TTCGTGGGCGGYAACTGG	64	[24]
		Tig_F2^c^	GCCTACATCACGGAGCARA		[24]
		G3PDH_For^c^	TCGCYATCAACGGMTTCGG		[24]
		Tig_Rev^c^	CCGAAKCCGTTGATRGCGA		[24]
		G3PDH_Rev	GCCCCACTCRTTGTCRTACCAC		[24]
*Mitochondrial*					
**Cox2+Cox Spacer**	Ia:7625–	FM35	CAGAACCTTGGCAATTAGG	54	[99]
(Cytochrome c oxidase subunit 2+spacer)	8537	FM82^c^	TTGGCAATTAGGTTTTCAAGATCC		[100]
		FM80^c^	AATATCTTTATGATTTGTTGAAA		[100]
		Phy10b	GCAAAAGCACTAAAAATTAAATATAA		[101]
**Nad9+Nad Spacer**	Ia:10511–	Nad9-F	TACAACAAGAATTAATGAGAAC	61	[39]
(NADH dehydrogenase subunit 9+spacer)	11167	Nad9-R	GTTAAAATTTGTACTACTAACAT		[39]
**RPS10**	Ia:19147–	Prv9-F	GTATACTCTAACCAACTGAGT	59	[39]
(Ribosomal protein S10)	19473	Prv9-R	GTTGGTTAGAGTAAAAGACT		[39]
**SecY**	Ia:29598–	SecY-F	TCTATCGTGTTTACCAATTTC	61	[100]
(SecY-independent transporter protein)	30344	SecY-R	TAACAAATGGATCTTCTTTAAAA		[100]

T_a_ – primer annealing temperature during amplification.

a ) Supercontig location or transcript number from *Phytophthora infestans* T30-4 genome (nuclear) or Ia haplotype (mitochondrial), Broad Institute (http://www.broadinstitute.org/annotation/genome/phytophthora_infestans/MultiHome.html).

b ) Reference sequence from *P. infestans* (NCBI database).

c ) Primers used for sequencing only.

d ) Touchdown amplification protocol also used (see Methods).

### Sequence Heterozygosity and Haplotype Diversity

**Figure 1:**
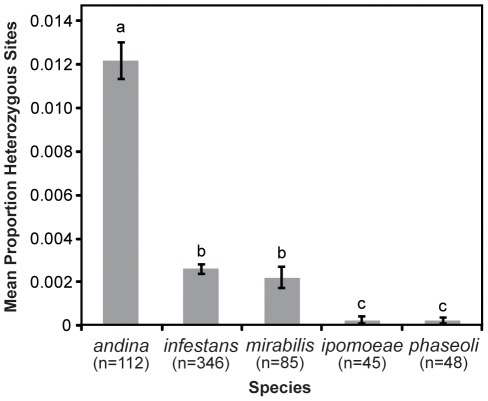
Proportion (mean ±1 SE) of heterozygous sites for each species within *Phytophthora* Clade 1C across fifteen nuclear loci. Lowercase letters above bars indicate significant differences as detected by Tukey's HSD test (*P*<0.001). n = number of individual sequences per species included in analysis.

Sequences were generated from multiple isolates (between 3 and 32) of Clade 1C species, and from single isolates of the remaining Clade 1 species (Table 2). Sequence heterozygosity in the six mitochondrial loci was negligible; only a single heterozygous site was identified out of more than 180,000 bases, and was resolved with additional sequencing. Higher levels of sequence heterozygosity were observed in the nuclear loci, revealing allelic differences in the diploid genome. Levels of heterozygosity differed significantly among the Clade 1C species (*F*_4,636_ = 100.137, *P*<0.0001; Table S2). *Phytophthora andina* showed significantly more heterozygous sites, up to six times more than the other heterothallic Clade 1C species (Figure 1). *Phytophthora infestans* and *P. mirabilis* showed similar levels of heterozygosity, which were both higher than the levels observed in the two homothallic species, *P. ipomoeae* and *P. phaseoli* (Figure 1).

**Table 2:** *Phytophthora* isolates used in this study.

Clade/Subclade	*Phytophthora* species^a^	Isolate Identification		Isolate Origins			Mating Type^f^
		Local^b^	International^c^	Host	Country	Date	
1	*P. nicotianae*	P6303		*Grammatophyllum* sp.	Indonesia	1989	A2
1a	*P. cactorum*	P0714	ATCC10091 CBS231.30	*Syringa vulgaris*	The Netherlands	1930^d^	Ho
	*P. hedraiandra*	P11056		*Rhododendron* sp.	USA	2006^e^	Ho
	*P. idaei* (T)	P6767	CBS971.95 IMI313728	*Rubus idaeus*	UK	1987	Ho
	*P. pseudotsugae* (T)	P10339	IMI331662	*Pseudotsuga menziesii*	USA	2003^e^	Ho
1b	*P. clandestina*	P3942	ATCC58715 CBS349.86	*Trifolium subterraneum*	Australia	1988^e^	Ho
	*P. iranica* (T)	P3882	ATCC60237 CBS374.72 IMI158964	*Solanum melongena*	Iran	1969	Ho
	*P. tentaculata*	P8497	CBS552.96	*Chrysanthemum leucanthemum*	Germany	1994^e^	Ho
1c	*P. andina*	P13365(T)		*Solanum brevifolium*	Ecuador	2001	A2
		P13539		*Solanum betaceum*	Ecuador	2002	A1
		P13576		*Solanum* sp. Anarrhichomenum complex	Ecuador	2002	A1
		P13642		*Solanum betaceum*	Ecuador	2003	A1
		P13648		*Solanum* sp. Anarrhichomenum complex	Ecuador	2003	A1
		P13655		*Solanum hispidium*	Ecuador	2003	A2
		P13660		*Brugmansia sanguinea*	Ecuador	2003	A1
		P13766		*Solanum betaceum*	Ecuador	2004	A1
		P13780		*Solanum hispidium*	Ecuador	2006^e^	A2
		P13803		*Solanum betaceum*	Ecuador	2004	A1
		P13821		*Solanum* sp. Anarrhichomenum complex	Ecuador	2004	A1
		P13865		*Solanum jugandifolium*	Ecuador	2005	A1
	*P. infestans*	P1305		*Solanum lycopersicon*	USA	1982	A1
		P1417		*Solanum tuberosum*	Isreal	1984	na
		P1847		*Solanum tuberosum*	UK	1983	A1
		P3681	ATCC64093	*Solanum tuberosum*	Mexico	1983	A1
		P3685		*Solanum tuberosum*	Mexico	1983	A1
		P6515		*Solanum tuberosum*	Peru	1989^e^	A1
		P6746		*Solanum tuberosum*	Poland	1989^e^	A2
		P6747		*Solanum tuberosum*	Poland	1989^e^	A1
		P6752		*Solanum tuberosum*	Mexico	1989^e^	A2
		P7035		*Solanum lycopersicon*	USA	1989	A1
		P9464		*Solanum tuberosum*	USA	1996	na
		P10052		*Solanum tuberosum*	USA	1998	A1
		P10053		*Solanum tuberosum*	Russia	1999^e^	A1
		P10110		*Solanum tuberosum*	USA	1994	A2
		P10112		*Solanum tuberosum*	USA	1994	A1
		P10124		*Solanum tuberosum*	USA	1994	A1
		P10157		*Solanum lycopersicon*	USA	1994	A1
		P10260		*Solanum lycopersicon*	Hungary	2002	A1
		P10650		*Solanum tuberosum*	Mexico	2004^e^	A1
		P11633		*Solanum lycopersicon*	Hungary	2005	SF
		P12021		*Solanum tuberosum*	Russia	2002	A2
		P12030		*Solanum tuberosum*	Russia	2003	A1
		P12038		*Solanum tuberosum*	Russia	2003	na
		P12043		*Solanum lycopersicon*	Russia	2003	A1
		P12044		*Solanum tuberosum*	Russia	2003	A1
		P12053		*Solanum tuberosum*	Russia	2001	A2
		P12102		*Solanum lycopersicon*	USA	2005	na
		P13198		*Solanum tuquerrense*	Ecuador	1998	A1
		P13346		*Solanum colombianum*	Ecuador	2001	A1
		P13626		*Solanum tuberosum*	Ecuador	2003	A1
		P13841		*Solanum habrochaires*	Ecuador	2004	A1
		P13873		*Solanum tuberosum*	Ecuador	2005	A1
	*P. ipomoeae*	P10225		*Ipomoea longipedunculata*	Mexico	1999	Ho
		P10226		*Ipomoea longipedunculata*	Mexico	1999	Ho
		P10227		*Ipomoea longipedunculata*	Mexico	1999	Ho
	*P. mirabilis*	P3005	ATCC64068 CBS150.88	*Mirabilis jalapa*	Mexico	1987^e^	A1
		P3006	ATCC64069	*Mirabilis jalapa*	Mexico	1987^e^	A2
		P3007	ATCC64070	*Mirabilis jalapa*	Mexico	1987^e^	A1
		P3009	ATCC64072	*Mirabilis jalapa*	Mexico	1987^e^	A1
		P3010	ATCC64073	*Mirabilis jalapa*	Mexico	1987^e^	A1
		P10228		*Mirabilis jalapa*	Mexico	2003^e^	na
		P10229		*Mirabilis jalapa*	Mexico	2003^e^	na
		P10230		*Mirabilis jalapa*	Mexico	2003^e^	na
		P10231		*Mirabilis jalapa*	Mexico	2003^e^	na
	*P. phaseoli*	P6609	CBS114106	*Phaseolus lunatus*	USA	1989	Ho
		P10145		*Phaseolus lunatus*	USA	2003^e^	Ho
		P10150		*Phaseolus lunatus*	USA	2003^e^	Ho
		P11082	CBS120373	*Phaseolus lunatus*	USA	2003	Ho
2	*P. capsici*	P0253	ATCC46012	*Theobroma cacao*	Mexico	1964	A1

a ) Type isolate (T).

b ) Local identification numbers from the World Oomycete Genetic Resource Collection, University of California-Riverside.

c ) International identification numbers from American Type Culture Collection (ATCC); Centraalbureau voor Schmmelcultures, The Netherlands (CBS); CABI Biosciences, UK (IMI).

d ) Date culture was obtained by CBS.

e ) Date culture was obtained by the World Oomycete Genetic Resource Collection, University of California-Riverside.

f ) Abbreviations for mating type: homothallic (Ho), not available (na), self-fertile (SF).

Haplotypes were predicted computationally for the nuclear loci, and confirmed experimentally for *P. andina* and select isolates of *P. infestans* and *P. mirabilis* via cloning of PCR products. Among the Clade 1C species, the number of observed haplotypes within each dataset ranged from 5 to 24 in the nuclear loci, and from 5 to 8 in the mitochondrial loci; nucleotide diversity ranged from 8.1×10^−4^ to 1.7×10^−2^ in the nuclear loci, and from 1.8×10^−3^ to 8.8×10^−3^ in the mitochondrial loci (Table 3). The presence of two distinct nuclear haplotypes (labeled “A” and “B”) was consistent in eleven out of twelve *P. andina* isolates (isolate P13865 was homozygous for the “A” haplotype). All *P. andina* isolates were homozygous for the “A” haplotype of the homogentisate 1,2-dioxygenase nuclear locus. For the mitochondrial data, *P. andina* isolates grouping together as the “A” haplotype (P13539, P13576, P13660, P13766, P13865) were mating type A1, while those grouping together as the “B” haplotype (P13365, P13655, P13780) were mating type A2.

**Table 3:** Genetic diversity observed in *Phytophthora* Clade 1C species per locus.

Locus	L	N	Segregating Sites	Observed Haplotypes	Haplotype Diversity	Nucleotide Diversity	θ_W_ (per site)	θ_W_ (per sequence)
*Nuclear*								
LSU	1339	18	4	5	0.765	0.0009	0.0009	1.163
60SL10	456	116	19	16	0.856	0.0095	0.0078	3.567
ARP2/3	965	113	52	16	0.804	0.0095	0.0102	9.811
Beta-tubulin	1134	116	37	18	0.759	0.0056	0.0061	6.946
Enolase	1176	22	34	15	0.952	0.0087	0.0079	9.327
HGD68	603	114	30	11	0.573	0.0090	0.0094	5.651
ITS	775	108	12	10	0.376	0.0008	0.0030	2.284
P4P5K	1019	79	63	23	0.864	0.0132	0.0125	12.752
Pelota	739	84	51	14	0.766	0.0175	0.0138	10.196
PUA	627	106	54	24	0.919	0.0158	0.0165	10.313
Ras	552	105	31	10	0.790	0.0132	0.0108	5.931
Ras Intron	305	104	20	10	0.673	0.0167	0.0126	3.834
TigA	1597	20	44	10	0.905	0.0084	0.0078	12.405
TRP1	624	108	23	19	0.847	0.0079	0.0070	4.377
*Mitochondrial*								
Cox2	684	50	15	6	0.731	0.0046	0.0049	3.349
Cox2 Spacer	357	50	6	5	0.470	0.0023	0.0038	1.340
Nad9	558	50	8	5	0.387	0.0018	0.0032	1.786
Nad9 Spacer	299	50	11	8	0.740	0.0088	0.0082	2.456
RPS10	327	51	6	6	0.658	0.0028	0.0041	1.334
SecY	747	50	15	7	0.785	0.0058	0.0045	3.349

Note: HSP90 was excluded from this analysis due to low confidence values on computationally predicted haplotypes.

L – alignment length.

N – number of sequences included in analysis.

θ_W_ – Watterson's estimator.

All datasets were tested for evidence of recombination and violation of neutral evolution. Both *P. mirabilis* and *P. infestans* showed evidence for recombination in some nuclear loci (seven out of thirteen for *P. infestans*, two out of thirteen for *P. mirabilis*). There was no evidence for recombination in any of the nuclear loci for *P. ipomoeae*, *P. phaseoli*, or *P. andina* haplotype “B”; *P. andina* haplotype “A” showed recombination in one nuclear locus. No mitochondrial data showed evidence for recombination. Neutral evolution was rejected in three loci for *P. infestans* and one locus for *P. mirabilis*. For the protein-coding loci, sequences were separated into coding and non-coding regions, and retested. Loci showing evidence of recombination or violation of neutral evolution were not included in the estimation of species trees.

### Phylogenetic Analysis

**Figure 2:**
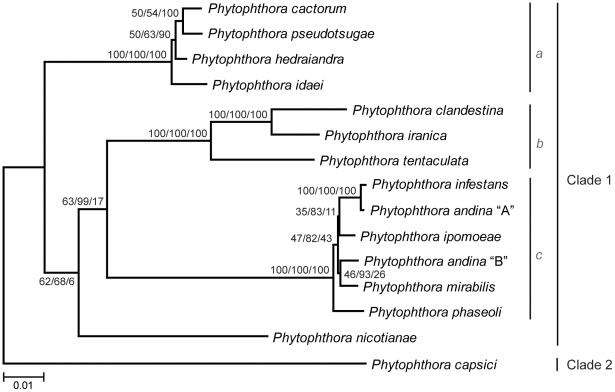
Phylogeny of *Phytophthora* Clade 1 based on eighteen loci (twelve nuclear, six mitochondrial; 14,792 basepairs). ML branch lengths are shown. Numbers on nodes represent bootstrap support values for ML (left) and MP (right), and Bayesian Posterior Probabilities as percentages (middle). All support values are shown for each analysis. Three nuclear loci (ARP2/3, HGD, Pelota) were excluded from the concatenation due to missing data for some Clade 1 species.

The Clade 1 phylogeny reconstructed from a concatenation of eighteen loci (Figure 2) was similar to previous studies [24], [25], [26]. Significant bootstrap and Bayesian posterior probability support were found for the division of Clade 1 into three subclades; A, B, and C. Our analyses suggested a closer relationship between subclades B and C, however the position of *P. nicotianae* remained unresolved, as in previous studies [24], [25], [26]. Within Clade 1C, a close relationship between *P. andina* haplotype “A” and *P. infestans* was well supported, but all other relationships among the species were unresolved.

**Figure 3:**
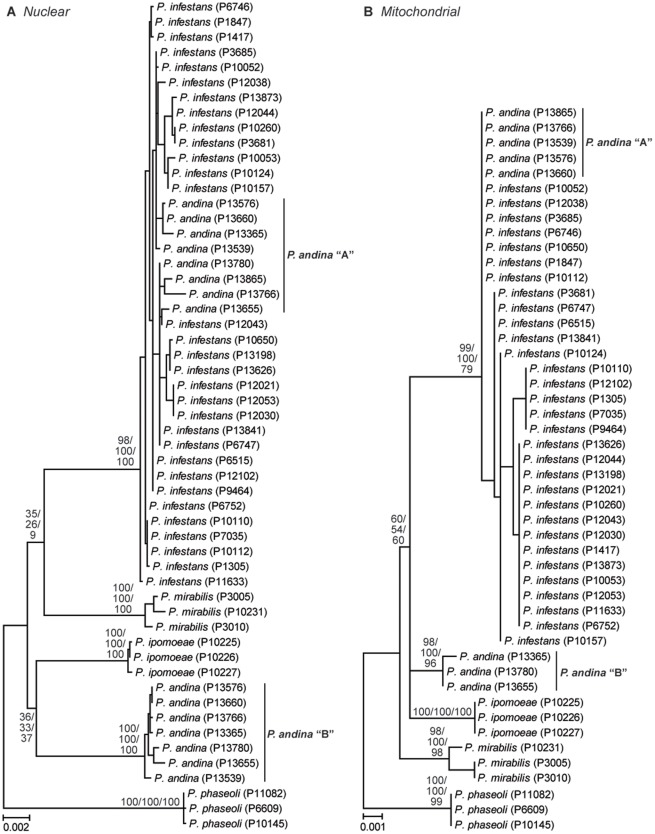
Phylogeny of *Phytophthora* Clade 1C based on eleven nuclear (A; 8018 bps) and six mitochondrial loci (B; 3035 basepairs). ML branch lengths are shown. Numbers on nodes represent bootstrap support values for ML (top) and MP (bottom), and Bayesian Posterior Probabilities as percentages (middle). All support values are shown for each analysis. Accession numbers from the World Oomycete Genetic Resource Collection are shown next to species names. Four nuclear loci (LSU, Enolase, HSP90, TigA) were excluded from the concatenation due to missing data for several Clade 1C isolates.

In our expanded analysis with multiple isolates per species, both the nuclear and mitochondrial concatenations showed *P. andina* haplotype “A” embedded within isolates of *P. infestans* (Figure 3A, B). Sequences of *P. andina* haplotype “B” formed a distinct, monophyletic lineage in both datasets. Aside from species monophyly, all other relationships were unresolved. In the nuclear data, weak support was found for a grouping of *P. ipomoeae* with *P. andina* haplotype “B”, as well as *P. mirabilis* with *P. infestans*+*P. andina* haplotype “A” (Figure 3A). In the mitochondrial data, moderate support existed for a basal position of *P. mirabilis* to *P. infestans*, *P. andina*, and *P. ipomoeae* (Figure 3B). *Phytophthora phaseoli* was consistently found to be the basal member of Clade 1C when *P. nicotianae* was used as an outgroup (data not shown), and was thus used to root the Clade 1C phylogeny.

### Species Tree Estimation

To avoid any potential bias from uneven sampling, up to six sequences representing haplotypes were selected from each lineage for species tree estimation. For most nuclear loci, only six sequences were available for *P. ipomoeae* and *P. phaseoli*, with each species typically displaying a single haplotype. For *P. mirabilis* and *P. infestans*, sequences were chosen to reflect the haplotype diversity and frequency within each species. Six sequences were chosen for both the “A” and “B” haplotypes of *P. andina*. For the mitochondrial data, each lineage was represented by 3–6 sequences. Eight nuclear loci showed no evidence of recombination or non-neutral evolution, and were therefore used in the species tree analyses; estimates of nucleotide diversity for the six-haplotype datasets were similar to estimates from the complete datasets (Table S3).

**Figure 4:**
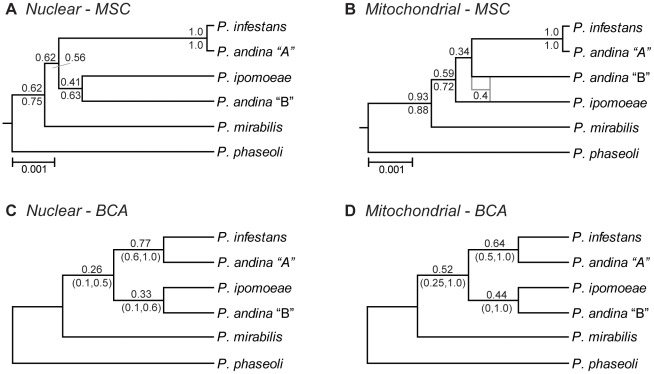
Species tree estimates from nuclear and mitochondrial datasets for *Phytophthora* Clade 1C. **A, B**: Topologies estimated under the multispecies coalescent model (MSC); numbers on nodes represent posterior probabilities from *Beast under a strict molecular clock model (above) and a relaxed lognormal clock model (below). **C, D**: Primary concordance trees estimated by Bayesian concordance analysis (BCA); numbers on nodes represent sample-wide clade concordance factors (above) and 95% credibility intervals (below).

Under the multispecies coalescent approach, two methods of species tree estimation were used for both the nuclear and mitochondrial datasets; the Bayesian method, *Beast [12]; and the maximum likelihood method, STEM [10]. For the nuclear data, the *Beast analysis showed good convergence with 100 million generations, and a majority of parameters had ESS values >200 (two model rate parameters in one locus showed low ESS values). The topologies of the two YPT1 loci were linked *a priori* as it is unlikely that these two regions freely recombine. The resulting species tree strongly supported *P. andina* haplotype “A” with *P. infestans*, and showed moderate support for a relationship between *P. andina* haplotype “B” and *P. ipomoeae* (Figure 4A). The topology and support values were robust to changes in the tree prior (Yule versus birth-death process) and molecular clock model (strict versus relaxed). The individual gene trees and rate values estimated under a strict clock model in *Beast were then used as the input for STEM (Table 4); the resulting maximum likelihood species tree had an identical topology to the *Beast estimate. However, a search of the fifteen highest likelihood trees revealed a set of topologies with unresolved relationships among *P. ipomoeae*, *P. mirabilis*, and *P. andina* haplotype “B” with similar likelihood values (Table S4).

**Table 4:** Individual gene trees estimating from *Beast.

Locus	L (VS)	Model	Topology	Mean lnL	Clock Rate
*Nuclear*					
60SL10	456 (15)	TrN+I	(((((infestans, andinaA), ipomoeae), andinaB), mirabilis), phaseoli)	−771.43	2.80
ARP2/3 (introns)	372 (30)	GTR+I	((((infestans, andinaA), (ipomoeae, andinaB)), mirabilis), phaseoli)	−727.73	3.82
Beta-tubulin	822 (21)	TrN	(((((infestans, andinaA), ipomoeae), andinaB), mirabilis), phaseoli)	−1291.43	1.00
ITS	845 (11)	HKY	((((infestans, andinaA), ipomoeae), andinaB), mirabilis), phaseoli)	−1274.50	0.46
Pelota	744 (50)	TrN+I	(((((infestans, andinaA), ipomoeae), andinaB), mirabilis), phaseoli)	−1418.31	3.18
Ras (coding)	325 (3)	HKY	((((infestans, andinaA), (ipomoeae, andinaB)), mirabilis), phaseoli)	−476.81	0.35
Ras Intron	308 (22)	HKY+I		−613.64	3.25
TRP1 (coding)	467 (10)	HKY	((((infestans, andinaA), (ipomoeae, andinaB)), mirabilis), phaseoli)	−722.97	0.79
*Mitochondrial*					
Cox2	684 (15)	HKY	(((((infestans, andinaA), andinaB), ipomoeae), mirabilis), phaseoli)	−972.80	1.00
Cox2 spacer	392 (8)	HKY		−519.30	0.98
Nad9	567 (7)	HKY	(((((infestans, andinaA), andinaB), ipomoeae), mirabilis), phaseoli)	−729.39	0.63
Nad9 spacer	299 (11)	HKY		−426.55	2.83
RPS10	327 (5)	HKY	(((((infestans, andinaA), andinaB), ipomoeae), mirabilis), phaseoli)	−384.54	0.72
SecY	747 (15)	HKY	((((infestans, andinaA), (andinaB, ipomoeae)), mirabilis), phaseoli)	−854.61	0.97

L – alignment length.

VS – number of variable sites within alignment.

For the mitochondrial data, the topologies of the six loci were linked *a priori* in *Beast to reflect the non-recombining nature of the mitochondrial genome. The analysis showed good convergence with 100 million generations, and all parameters had ESS values >200. The species tree estimated for the mitochondrial data under a strict clock model showed *P. andina* haplotype “B” as more closely related to the group of *P. infestans*+*P. andina* haplotype “A” than to *P. ipomoeae* (Figure 4B). However, the topology estimated under a relaxed lognormal clock model was identical to the nuclear results, with *P. andina* haplotype “B” grouping with *P. ipomoeae*. In order to estimate the maximum likelihood species tree, a second *Beast analysis was performed under a strict molecular clock model with the topologies of the two *cox* loci and the two *nad* loci linked *a priori*; the individual gene trees and rate values were then used as input for STEM (Table 4). The maximum likelihood species tree showed an identical topology to the strict clock *Beast estimate, although a search of the fifteen highest likelihood trees again revealed a set of unresolved topologies with similar likelihood values (Table S4). These results are presented with the caveat that analyzing individual gene trees from the mitochondrial dataset violates the assumption of free recombination among loci.

The primary concordance trees estimated for both the nuclear and mitochondrial datasets under the Bayesian concordance approach showed the same topology as the species tree estimated for the nuclear data under the multispecies coalescent approach (Figure 4C, D). These results were robust to differing values of alpha (α), the parameter controlling the prior probability on gene tree discordance, and population trees based on quartet analysis were identical to the primary concordance trees. For the nuclear dataset, there was equivalent support for a position of *P. mirabilis* sister to *P. ipomoeae*+*P. andina* haplotype “B” (concordance factor 0.26; 95% CI 0.0, 0.5); other relationships conflicting with the primary concordance tree showed lower concordance factors (Table S5). As in the maximum likelihood analysis under the multispecies coalescent model, the mitochondrial dataset violates the assumption of free recombination among loci; we therefore present the Bayesian concordance results with this caveat.

## Discussion

The availability of new methods for the explicit estimation of species trees allows us to test hypotheses about speciation while accommodating heterogeneity in the evolutionary process. Here we have used three approaches to determine the relationships among the five foliar pathogens in *Phytophthora* Clade 1C. Our concatenation-based analyses were unable to resolve the relationships among species despite the use of multilocus data from both the nuclear and mitochondrial genomes (Figure 2) and multiple isolates per species (Figure 3). These “supergene” methods have been criticized because they assume a single underlying topology across all loci; this condition is unlikely to be true due to processes such as incomplete lineage sorting, especially when internal branches are short [5], [40]. We therefore used two methods implementing the multispecies coalescent approach, which assumes incomplete lineage sorting is the main source of discordance among gene trees [6]. Both the Bayesian method, *Beast [12], and the maximum likelihood method, STEM [10], estimated the same species tree (Figure 4A, B). The nuclear and mitochondrial topologies differed slightly, but both datasets supported a close relationship among *P. infestans*, *P. andina*, and *P. ipomoeae*. While support values on the species trees generated by *Beast may seem low as compared to those typically obtained in concatenation-based studies of gene trees (e.g., bootstrap support), it is unclear how to compare these related but distinct statistical measures of confidence; low support values in coalescent-based analyses can result if incomplete lineage sorting is not the only source of discordance between gene trees and the species tree [7]. Similarly, the occurrence of several unresolved topologies with comparable likelihood values in our STEM analyses is not unexpected given the short internal branch lengths in the gene trees, a condition shown to limit the accuracy of this method [41].

We have also used Bayesian concordance analysis [16] to estimate the dominant phylogenetic history of the Clade 1C species. The primary concordance topologies estimated from the nuclear and mitochondrial datasets were both identical to the species tree estimated from the nuclear data under the multispecies coalescent model (Figure 4C, D). It is important to note that concordance factors are not equivalent to confidence values generated in phylogenetic analyses; concordance factors simply represent the proportion of the genome (or sample) for which a split is recovered [15], [42]. Thus concordance factors can be influenced by evolutionary processes such as reticulation (e.g., gene flow), as well as analytical issues such as flat posterior distributions on gene trees [15]. Other empirical studies have also observed low concordance factors for nodes that were supported in species tree estimates [2], [43]. The population trees estimated under a quartet-based consensus method [44] were identical to the primary concordance trees, further suggesting that the resulting topology reflects the dominant phylogenetic history for Clade 1C, despite low concordance factors.

As in previous studies [29], [32], [33], our results support a hybrid origin for *P. andina*, with *P. infestans* representing the parental lineage of the “A” haplotype described here. The “B” haplotype of *P. andina* represents an independent evolutionary lineage within Clade 1C which is more closely related to *P. ipomoeae* than to either *P. infestans* or *P. mirabilis*. In addition, the presence of two distinct mitochondrial haplotypes, which segregated with mating type and are inherited uniparentally, suggests that multiple hybridization events may have taken place during the formation of the hybrid *P. andina*. While coalescent-based methods have been proposed to estimate species trees in the presence of hybridization [45], [46], [47], our dataset appeared to violate the model assumptions since no free-living parental lineage is known for the “B” haplotype of *P. andina*. While it is possible that this unknown parental species exists in nature but has not yet been collected [32], it is equally possible that the hybrid *P. andina* has replaced or outcompeted the parental lineage on their shared hosts [48]. Hybridization likely equipped the new lineage with novel combinations of effectors and other plant-induced pathogenicity genes; it has been shown that these gene families reside in repeat-rich, gene-sparse regions of the *P. infestans* genome, where they evolve rapidly and likely play an important role in adaptation to new hosts [37].

Other interspecific hybrids of *Phytophthora* have been described from natural environments. *Phytophthora cactorum* has been shown to be involved in several hybridization events with other closely related members of Clade 1, particularly in greenhouse settings [49], [50], [51], [52]. *Phytophthora alni* was first isolated in the early 1990s from dying alder trees in the UK [53], and has since been found across Europe [54]. Evidence from both nuclear and mitochondrial data suggests that *P. alni* subsp. *alni* is an allopolyploid hybrid of the other two described subspecies, *P. alni* subsp. *uniformis* and *P. alni* subsp. *multiformis* [55], [56], [57]; the hybrid subsp. *alni* is also more aggressive on alder [58]. While the subspecies of *P. alni* show variations in ploidy (near tetraploidy in subsps. *alni* and *multiformis*, near diploidy in subsp. *uniformis*; [55], [56], [57]), little is known about the ploidy level of *P. andina*. Our results consistently showed one to two haplotypes per isolate, suggesting that *P. andina* may be diploid and the product of homoploid hybrid speciation. Although *P. andina* is heterothallic, only clonal lineages have so far been described [29]. Species produced via recombinatorial hybridization often show reduced fertility due to differences in the position of chromosomal translocations in the parental lineages; chromosomal imbalances also reinforce post-zygotic reproductive barriers, preventing introgression with the parental lineages [48], [59], [60]. Host specialization, which may be occurring in one clonal lineage of *P. andina* [29], may also be providing pre-zygotic, ecological barriers to introgression with the parental lineages [61].

*Phytophthora* Clade 1C likely originated in the New World tropics, as this is the center of origin and/or diversity for all the major hosts [62], [63], [64], [65]. The Andes of South America have been proposed as the origin for *P. infestans* as this is a center of diversity for the Solanaceae [66], as well as the center of domestication for potato [67] and possibly tomato [68]. A South American origin has also been suggested based on historical observations of late blight in the indigenous potato-growing regions of Peru and Bolivia [69]. A recent coalescent-based analysis of nuclear and mitochondrial loci suggested that the oldest mutations found in *P. infestans* populations originated in South America [70]. Others, however, have suggested that the presence of a genetically diverse, sexually reproducing population, as well as the sister species *P. ipomoeae* and *P. mirabilis*, in the highland regions of central Mexico indicates a likely origin there [20]. The occurrence of resistance genes in wild, endemic potato species has also been argued as evidence for an extensive period of host-pathogen co-evolution in central Mexico [20], [71]. Our data may be more in line with a Mexican origin for Clade 1C due to the basal position of *P. mirabilis*. However, paleoecological changes over the past ∼10 million years, such as the final uplift of the Andes, the closing of the Isthmus of Panama, and glaciations during the Pleistocene [72], may have significantly altered the distributions of both hosts and pathogens. While molecular clock methods have been used to estimate the time of origin for several Neotropical groups (e.g., [73], [74], [75]), few reliable calibrations exist within the Oomycota fossil record to calibrate coalescent-based speciation times with absolute geologic time [76]. In addition, our datasets typically contained only a single haplotype from *P. ipomoeae* and *P. phaseoli*, making our estimates of population size and speciation times less reliable [12]. Additional data will be needed to determine when *P. infestans*, and Clade 1C in general, originated; this in turn may provide additional insight into the conditions leading to the hybrid origin of *P. andina*.

## Methods

### Sequence generation

Cultures were maintained and DNA was extracted as previously described [24]. PCR conditions for nuclear loci were as follows: 1× PCR buffer with a final MgCl_2_ concentration of 2.5 mM, 200 µM dNTPs, 0.2 µM of each primer, one unit of *Taq* polymerase, and ∼5 ng template DNA. Thermal cycling protocols used an initial denaturation step at 94°C for two minutes; 35 cycles of 94°C for 30 seconds, locus-specific annealing temperature for 30 seconds, 72°C extension for 1 minute (2 minutes for amplicons >1 kb); and a final extension at 72°C for five minutes. A touchdown protocol was used for some templates; an initial annealing temperature of 65°C was lowered by 1°C per cycle for 10 cycles, followed by an additional 30 cycles at an annealing temperature of 56°C. For mitochondrial loci, PCR reactions contained 1× amplification buffer with a final MgCl_2_ concentration of 3 mM, 100 µM dNTPs, 0.5 µM of each primer, and one unit of AmpliTaq (Applied Biosystems). Thermal cycling protocols used an initial denaturation step at 95°C for 3 minutes; 35 cycles of 95°C for one minute, locus-specific annealing temperature for 1 minute, 72°C extension for 2 minutes; and a final extension at 72°C for five minutes.

PCR products were visualized on a 1% agarose gel to confirm amplicon size. An enzymatic purification protocol was used following the manufacturer's instructions (ExoSAP-IT, Affymetrix), and products were sequenced using the BigDye system (version 3.1 dye terminators; Applied Biosystems) run on an ABI 3730XL DNA Analyzer at the Pennsylvania State University's Huck Institute Nucleic Acids Facility. ABI trace files were analyzed using Sequencher version 4 (GeneCodes); bases with overlapping peaks of equivalent size in the electropherograms were considered heterozygous and coded according to IUPAC convention. Sequences were aligned for each locus with ClustalX [77] and edited manually when necessary in MEGA version 4.0 [78]. *Phytophthora infestans* genome sequences of each locus, plus the predicted transcripts, were included in alignments of nuclear loci to identify exon/intron boundaries. Any available EST sequences from Clade 1 species were also obtained via BLAST [79] on the NCBI website (http://www.ncbi.nlm.nih.gov/) to confirm predicted exon/intron boundaries. All alignment files are available from the author (JEB).

### Analysis of Heterozygosity

The proportion of heterozygous sites per locus was compared among species via analysis of variance using a general linear model in SPSS version 19 (IBM). A fully factorial model was fitted with species and locus as fixed factors. Proportion values were arcsine, square root transformed prior to analysis to satisfy normality assumptions. Because loci differed in total length (315–1639 basepairs), a weighted least-square analysis was performed, using total sequence length for each locus as weights in the model. Post-hoc comparisons of heterozygosity among Clade 1C species were obtained using the Tukey HSD test.

### Haplotype determination

Haplotypes were computationally predicted for ungapped genotypic alignments using the programs Arlequin version 3.1 (EM zipper option) [80], GERBIL [81], HAPINFERX [82], HaploRec version 2.3 [83], and PHASE version 2.1 [84]. The CVhaplot package version 2.0 [85] was used to generate the input files for each program, as well as calculate the consensus haplotypes from the outputs using a consensus vote approach [86]. Predicted haplotypes were then realigned with the original sequence data and the experimentally determined haplotypes (see below) to reestablish indel polymorphisms. DnaSP [87] was used to calculate DNA polymorphism statistics for each haplotype dataset.

Haplotypes were also experimentally confirmed for *P. andina* and some other Clade 1C isolates via cloning. PCR products were obtained as described above but with the use of a high-fidelity *Taq* polymerase (FideliTaq; Affymetrix). Amplicons were purified using the QIAquick PCR purification kit and cloned using the T/A-based PCR cloning kit (QIAGEN). Transformed cells were selected for a new round of PCR amplification using plasmid-specific primers (Sp6, T7) and a high-fidelity *Taq* polymerase; products were cleaned and sequenced as described above.

### Phylogenetic analyses

ModelTest version 3.7 [88] was used to identify the appropriate evolutionary model for each dataset, according to the Akaike Information Criterion. Maximum likelihood analyses were performed in GARLI version 2.0 [89] with an initial search (two replicates) used to estimate the model parameters; these parameters were then fixed for a bootstrap analysis with 1000 replicates. A majority-rule consensus of the bootstrap replicates was calculated in PAUP version 4b10 [90] or Consense in the Phylip package [91]. Bayesian analyses were performed in MrBayes version 3.1 [92] with two searches run simultaneously for at least two million generations. Flat Dirichlet priors were used for the nucleotide base frequencies and the model rate parameters. Uniform priors between zero and one were used for the gamma shape parameter and the proportion of invariable sites. Three heated chains (temperature 0.2) and one cold chain were used in each search. Tracer version 1.5 [93] was used to evaluate mixing and convergence, and to estimate the appropriate burn-in period. The majority-rule consensus was then calculated after removing the first 10% of generations as burn-in. Maximum parsimony analyses were performed in DNAPars in the Phylip package [91]; bootstrap replicates were generated using SeqGen (500–1000 replicates). The majority-rule consensus tree was generated using Consense.

### Species Tree Estimation

Individual datasets for each locus were limited to six sequences (representing haplotypes) per lineage to avoid any potential bias from uneven sampling. ModelTest version 3.7 was used to identify the appropriate evolutionary model for each dataset, and DnaSP was used to calculate DNA polymorphism statistics. BEAUTi version 1.6.1 [94] was used to create the XML-formatted input files for *Beast [12]; species were indicated for each sequence under the Traits tab, and the evolutionary model was specified for each locus. All mitochondrial loci were analyzed under an HKY model in the *Beast analyses due to low convergence when more complex models were used. Evolutionary rates were estimated by fixing one locus at a value of 1.0 (beta-tubulin for the nuclear dataset, *cox2* for the mitochondrial dataset). Individual gene trees were linked *a priori* as appropriate, and both the Yule and birth-death species tree priors were tested. Each analysis was performed twice, once under a strict molecular clock model and once under a relaxed lognormal clock model. All datasets were run for 100 million generations in *Beast, sampling every 10,000 generations; log files were evaluated in Tracer version 1.5. Both the species tree and individual gene trees were calculated using TreeAnnotator version 1.6.1 [95] with a burn-in of 1000 trees.

The individual gene trees and rates estimated under a strict clock model in *Beast were used as input for STEM version 2.0 [10]. The average value of Watterson's theta (per site) estimated for each locus in DnaSP was used as the theta parameter in STEM (0.01 for the nuclear data, 0.006 for the mitochondrial data). Default settings were used for beta (0.0005) and the search parameters. Each dataset was analyzed twice, once to determine the maximum likelihood tree with branch lengths (run = 1) and once to estimate the fifteen highest likelihood trees (run = 2).

For the Bayesian concordance analysis, each dataset was first analyzed in MrBayes version 3.1 with two searches run simultaneously for two million generations, sampling every 1000 generations. All other settings were identical to the Bayesian phylogenetic analysis (see above), except that the individual lineages were constrained to be monophyletic. For the mitochondrial data, the two *cox* loci and the two *nad* loci were combined into single datasets. Tree files were summarized for each locus using the program mbsum with a burn-in of 200 trees. The Bayesian concordance analysis was then performed in the program BUCKy version 1.4 [44] with four independent runs, each with one million generations and four chains. Three values of the alpha parameter were tested (0.1, 1.0, 10.0); an additional value (0.01) was also tested for the mitochondrial data to emphasize the expected concordance among loci (since the mitochondrial genome does not recombine). Default settings were used for all other parameters. The primary concordance topology and clade concordance factors with 95% credibility intervals were determined for both the nuclear and mitochondrial datasets.

## Supporting Information

Table S1 **NCBI accession numbers for sequences analyzed in this study.**
(XLS)

Table S2 **Tests of between-subjects effects, weighted least squares regression (weighted by locus length).**
(DOC)

Table S3 **Genetic diversity of** ***Phytophthora*** **Clade 1C species estimated from six-haplotype datasets.**
(XLS)

Table S4 **Alternative maximum likelihood topologies for nuclear and mitochondrial datasets.**
(DOC)

Table S5 **Minor splits and concordance factors found in the Bayesian Concordance Analysis.**
(DOC)

## References

[1] Clark AG, *Drosophila* Genome Consortium (2007). Evolution of genes and genomes on the *Drosophila* phylogeny.. Nature.

[2] Cranston KA, Hurwitz B, Ware D, Stein L, Wing RA (2009). Species trees from highly incongruent gene trees in rice.. Systematic Biology.

[3] Rokas A, Williams BL, King N, Carroll SB (2003). Genome-scale approaches to resolving incongruence in molecular phylogenies.. Nature.

[4] Edwards SV, Liu L, Pearl DK (2007). High-resolution species trees without concatenation.. Proceedings of the National Academy of Sciences USA.

[5] Kubatko LS, Degnan JH (2007). Inconsistency of phylogenetic estimates from concatenated data under coalescence.. Systematic Biology.

[6] Degnan JH, Rosenberg NA (2009). Gene tree discordance, phylogenetic inference and the multispecies coalescent.. Trends in Ecology & Evolution.

[7] Edwards SV (2009). Is a new and general theory of molecular systematics emerging?. Evolution.

[8] Maddison WP (1997). Gene trees in species trees.. Systematic Biology.

[9] Liu L, Yu L, Kubatko L, Pearl DK, Edwards SV (2009). Coalescent methods for estimating phylogenetic trees.. Molecular Phylogenetics and Evolution.

[10] Kubatko LS, Carstens BC, Knowles LL (2009). STEM: species tree estimation using maximum likelihood for gene trees under coalescence.. Bioinformatics.

[11] Ence DD, Carstens BC (2011). SpedeSTEM: a rapid and accurate method for species delimitation.. Molecular Ecology Resources.

[12] Heled J, Drummond AJ (2010). Bayesian inference of species trees from multilocus data.. Molecular Biology and Evolution.

[13] Liu L (2008). BEST: Bayesian estimation of species trees under the coalescent model.. Bioinformatics.

[14] Liu L, Yu L, Pearl DK, Edwards SV (2009). Estimating species phylogenies using coalescence times among sequences.. Systematic Biology.

[15] Baum DA (2007). Concordance trees, concordance factors, and the exploration of reticulate genealogy.. Taxon.

[16] Ane C, Larget B, Baum DA, Smith SD, Rokas A (2007). Bayesian estimation of concordance among gene trees.. Molecular Biology and Evolution.

[17] Zadoks JC (2008). On the political economy of plant disease epidemics.

[18] Moskin J (2009). Outbreak of fungus threatens tomato crop..

[19] Savary S, Nelson A, Sparks AH, Willocquet L, Duveiller E (2011). International agricultural research tackling the effects of global and climate changes on plant diseases in the developing world.. Plant Disease.

[20] Grunwald NJ, Flier WG (2005). The biology of *Phytophthora infestans* at its center of origin.. Annual Review of Phytopathology.

[21] Erwin DC, Ribeiro OK (1996). Phytophthora Diseases Worldwide.

[22] Haverkort A, Struik P, Visser R, Jacobsen E (2009). Applied biotechnology to combat late blight in potato caused by *Phytophthora infestans*.. Potato Research.

[23] Fry WE, Grunwald NJ, Cooke D, McLeod A, Forbes GA, Lamour K, Kamoun S (2009). Population genetics and population diversity of *Phytophthora infestans*.. Oomycete Genetics and Genomics: Diversity, Interactions, and Research Tools.

[24] Blair JE, Coffey MD, Park S-Y, Geiser DM, Kang S (2008). A multi-locus phylogeny for *Phytophthora* utilizing markers derived from complete genome sequences.. Fungal Genetics and Biology.

[25] Cooke D, Drenth A, Duncan J, Wagels G, Brasier C (2000). A molecular phylogeny of *Phytophthora* and related Oomycetes.. Fungal Genetics and Biology.

[26] Kroon LPNM, Bakker FT, van den Bosch GBM, Bonants PJM, Flier WG (2004). Phylogenetic analysis of *Phytophthora* species based on mitochondrial and nuclear DNA sequences.. Fungal Genetics and Biology.

[27] Galindo J, Hohl HR (1985). *Phytophthora mirabilis*, a new species of *Phytophthora*.. Sydowia.

[28] Flier WG, Grunwald NJ, Kroon LPNM, van den Bosch GBM, Garay-Serrano E (2002). *Phytophthora ipomoeae* sp. nov., a new homothallic species causing leaf blight on *Ipomoea longipedunculata* in the Toluca Valley of central Mexico.. Mycological Research.

[29] Oliva RF, Kroon LPNM, Chacón G, Flier WG, Ristaino JB (2010). *Phytophthora andina* sp. nov., a newly identified heterothallic pathogen of solanaceous hosts in the Andean highlands.. Plant Pathology.

[30] Adler NE, Erselius LJ, Chacon MG, Flier WG, Ordonez ME (2004). Genetic diversity of *Phytophthora infestans* sensu lato in Ecuador provides new insight into the origin of this important plant pathogen.. Phytopathology.

[31] Ordonez ME, Hohl HR, Velasco JA, Ramon MP, Oyarzun PJ (2000). A novel population of *Phytophthora*, similar to *P. infestans*, attacks wild *Solanum* species in Ecuador.. Phytopathology.

[32] Goss EM, Cardenas ME, Myers K, Forbes GA, Fry WE (2011). The plant pathogen *Phytophthora andina* emerged via hybridization of an unknown *Phytophthora* species and the Irish potato famine pathogen, *P. infestans*.. PLoS ONE.

[33] Gomez-Alpizar L, Hu C-H, Oliva R, Forbes G, Ristaino JB (2008). Phylogenetic relationships of *Phytophthora andina*, a new species from the highlands of Ecuador that is closely related to the Irish potato famine pathogen *Phytophthora infestans*.. Mycologia.

[34] Cárdenas M, Tabima J, Fry WE, Grünwald NJ, Bernal A (2012). Defining species boundaries in the genus *Phytophthora*: the case of *Phytophthora andina*: A response to ‘*Phytophthora andina* sp. nov., a newly identified heterothallic pathogen of solanaceous hosts in the Andean highlands’ (Oliva et al., 2010).. Plant Pathology.

[35] Forbes GA, Ristaino JB, Oliva RF, Flier W (2012). A rebuttal to the letter to the editor concerning ‘Defining species boundaries in the genus *Phytophthora*: the case of *Phytophthora andina*’.. Plant Pathology.

[36] Haas BJ, Kamoun S, Zody MC, Jiang RHY, Handsaker RE (2009). Genome sequence and analysis of the Irish potato famine pathogen *Phytophthora infestans*.. Nature.

[37] Raffaele S, Farrer RA, Cano LM, Studholme DJ, MacLean D (2010). Genome evolution following host jumps in the Irish potato famine pathogen lineage.. Science.

[38] Chen Y, Roxby R (1996). Characterization of a *Phytophthora infestans* gene involved in vesicle transport.. Gene.

[39] Martin FN, Coffey MD (2012). Mitochondrial haplotype analysis for differentiation of isolates of *Phytophthora cinnamomi*.. Phytopathology.

[40] Pamilo P, Nei M (1988). Relationships between gene trees and species trees.. Molecular Biology and Evolution.

[41] Leache AD, Rannala B (2011). The accuracy of species tree estimation under simulation: a comparison of methods.. Systematic Biology.

[42] Ane C, Knowles LL, Kubatko L (2010). Reconstructing concordance trees and testing the coalescent model from genome-wide data sets.. Estimating Species Trees: Practical and Theoretical Aspects.

[43] Jacobsen F, Omland KE (2011). Species tree inference in a recent radiation of orioles (Genus *Icterus*): Multiple markers and methods reveal cytonuclear discordance in the northern oriole group.. Molecular Phylogenetics and Evolution.

[44] Larget BR, Kotha SK, Dewey CN, Ane C (2010). BUCKy: Gene tree/species tree reconciliation with Bayesian concordance analysis.. Bioinformatics.

[45] Meng C, Kubatko LS (2009). Detecting hybrid speciation in the presence of incomplete lineage sorting using gene tree incongruence: A model.. Theoretical Population Biology.

[46] Kubatko LS (2009). Identifying hybridization events in the presence of coalescence via model selection.. Systematic Biology.

[47] Yu Y, Than C, Degnan JH, Nakhleh L (2011). Coalescent histories on phylogenetic networks and detection of hybridization despite incomplete lineage sorting.. Systematic Biology.

[48] Schardl CL, Craven KD (2003). Interspecific hybridization in plant-associated fungi and oomycetes: a review.. Molecular Ecology.

[49] Bonants PJM, Hagenaar-de Weerdt M, Man in 't Veld WA, Baayen RP (2000). Molecular characterization of natural hybrids of *Phytophthora nicotianae* and *P. cactorum*.. Phytopathology.

[50] Man in 't Veld WA, de Cock AWAM, Summerbell RC (2007). Natural hybrids of resident and introduced *Phytophthora* species proliferating on multiple hosts.. European Journal of Plant Pathology.

[51] Man in 't Veld WA, Veenbaas-Rijks WJ, Ilieva E, de Cock AWAM, Bonants PJM (1998). Natural hybrids of *Phytophthora nicotianae* and *Phytophthora cactorum* demonstrated by isozyme analysis and random amplified polymorphic DNA.. Phytopathology.

[52] Hurtado-Gonzales OP, Aragon-Caballero LM, Flores-Torres JG, Man in't Veld W, Lamour KH (2009). Molecular comparison of natural hybrids of *Phytophthora nicotianae* and *P. cactorum* infecting loquat trees in Peru and Taiwan.. Mycologia.

[53] Brasier CM, Cooke DEL, Duncan JM (1999). Origin of a new *Phytophthora* pathogen through interspecific hybridization.. Proceedings of the National Academy of Sciences USA.

[54] Brasier CM, Kirk SA, Delcan J, Cooke DEL, Jung T (2004). *Phytophthora alni* sp. nov. and its variants: designation of emerging heteroploid hybrid pathogens spreading on *Alnus* trees.. Mycological Research.

[55] Ioos R, Andrieux A, Marcais B, Frey P (2006). Genetic characterization of the natural hybrid species *Phytophthora alni* as inferred from nuclear and mitochondrial DNA analyses.. Fungal Genetics and Biology.

[56] Ioos R, Barres B, Andrieux A, Frey P (2007). Characterization of microsatellite markers in the interspecific hybrid *Phytophthora alni* ssp. *alni*, and cross-amplification with related taxa.. Molecular Ecology Notes.

[57] Ioos R, Panabieres F, Industri B, Andrieux A, Frey P (2007). Distribution and expression of elicitin genes in the interspecific hybrid oomycete *Phytophthora alni*.. Applied and Environmental Microbiology.

[58] Brasier CM, Kirk SA (2001). Comparative aggressiveness of standard and variant hybrid alder phytophthoras, *Phytophthora cambivora* and other *Phytophthora* species on bark of *Alnus*, *Quercus* and other woody hosts.. Plant Pathology.

[59] Buerkle CA, Morris RJ, Asmussen MA, Rieseberg LH (2000). The likelihood of homoploid hybrid speciation.. Heredity.

[60] Mallet J (2007). Hybrid speciation.. Nature.

[61] Gross BL, Rieseberg LH (2005). The ecological genetics of homoploid hybrid speciation.. Journal of Heredity.

[62] Douglas NA, Manos PS (2007). Molecular phylogeny of Nyctaginaceae: taxonomy, biogeography, and characters associated with a radiation of xerophytic genera in North America.. American Journal of Botany.

[63] McDonald A (1991). Origin and diversity of Mexican Convolvulaceae.. Anales del Instituto de Biologia, Serie Botanica.

[64] Serrano-Serrano ML, Hernandez-Torres J, Castillo-Villamizar G, Debouck DG, Chacon Sanchez MI (2010). Gene pools in wild Lima bean (*Phaseolus lunatus* L.) from the Americas: Evidences for an Andean origin and past migrations.. Molecular Phylogenetics and Evolution.

[65] Olmstead RG, Bohs L, Migid HA, Santiago-Valentin E, Garcia VF (2008). A molecular phylogeny of the Solanaceae.. Taxon.

[66] Knapp S, Bohs L, Nee M, Spooner DM (2004). Solanaceae — a model for linking genomics with biodiversity.. Comparative and Functional Genomics.

[67] Spooner DM, McLean K, Ramsay G, Waugh R, Bryan GJ (2005). A single domestication for potato based on multilocus amplified fragment length polymorphism genotyping.. Proceedings of the National Academy of Sciences USA.

[68] Peralta IE, Spooner DM, Razdan MK, Mattoo AK (2007). History, origin, and early cultivation of tomato (Solanaceae).. Genetic Improvement of Solanaceous Crops, Vol 2: Tomato.

[69] Abad ZG, Abad JA (1997). Another look at the origin of late blight of potatoes, tomatoes, and pear melon in the Andes of South America.. Plant Disease.

[70] Gomez-Alpizar L, Carbone I, Ristaino JB (2007). An Andean origin of *Phytophthora infestans* inferred from mitochondrial and nuclear gene genealogies.. Proceedings of the National Academy of Sciences USA.

[71] Niederhauser JS, Lucas JA, Shattock RC, Shaw DS, Cooke LR (1991). *Phytophthora infestans*: the Mexican connection.. Phytophthora.

[72] Pennington R, Dick C, Hoorn C, Wesselingh F (2010). Diversification of the Amazonian flora and its relation to key geological and environmental events: a molecular perspective.. Amazonia, Landscape and Species Evolution: A Look into the Past: Wiley-Blackwell Publishing.

[73] Richardson JE, Pennington RT, Pennington TD, Hollingsworth PM (2001). Rapid diversification of a species-rich genus of Neotropical rain forest trees.. Science.

[74] Balke M, Gomez-Zurita J, Ribera I, Viloria A, Zillikens A (2008). Ancient associations of aquatic beetles and tank bromeliads in the Neotropical forest canopy.. Proceedings of the National Academy of Sciences USA.

[75] Weir JT, Price M (2011). Andean uplift promotes lowland speciation through vicariance and dispersal in *Dendrocincla* woodcreepers.. Molecular Ecology.

[76] Krings M, Taylor TN, Dotzler N (2011). The fossil record of the Peronosporomycetes (Oomycota).. Mycologia.

[77] Thompson JD, Gibson TJ, Plewniak F, Jeanmougin F, Higgins DG (1997). The CLUSTALX windows interface: flexible strategies for multiple sequence alignment aided by quality analysis tools.. Nucleic Acids Research.

[78] Tamura K, Dudley J, Nei M, Kumar S (2007). MEGA4: Molecular Evolutionary Genetics Analysis (MEGA) software version 4.0.. Molecular Biology and Evolution.

[79] Altschul SF, Madden TL, Schaffer AA, Zhang J, Zhang Z (1997). Gapped BLAST and PSI-BLAST: a new generation of protein database search programs.. Nucleic Acids Research.

[80] Excoffier L, Laval G, Schneider S (2005). Arlequin ver. 3.0: An integrated software package for population genetics data analysis.. Evolutionary Bioinformatics Online.

[81] Kimmel G, Shamir R (2005). GERBIL: Genotype resolution and block identification using likelihood.. Proceedings of the National Academy of Sciences USA.

[82] Clark AG (1990). Inference of haplotypes from PCR-amplified samples of diploid populations.. Molecular Biology and Evolution.

[83] Eronen L, Geerts F, Toivonen H (2006). HaploRec: efficient and accurate large-scale reconstruction of haplotypes.. BMC Bioinformatics.

[84] Stephens M, Smith NJ, Donnelly P (2001). A new statistical method for haplotype reconstruction from population data.. American Journal of Human Genetics.

[85] Huang Z-S, Zhang D-X (2010). CVhaplot: a consensus tool for statistical haplotyping.. Molecular Ecology Resources.

[86] Huang Z-S, Ji Y-J, Zhang D-X (2008). Haplotype reconstruction for scnp DNA: a consensus vote approach with extensive sequence data from populations of the migratory locust (*Locusta migratoria*).. Molecular Ecology.

[87] Rozas J, Sanchez-DelBarrio JC, Messeguer X, Rozas R (2003). DnaSP, DNA polymorphism analyses by the coalescent and other methods.. Bioinformatics.

[88] Posada D, Crandall KA (1998). MODELTEST: testing the model of DNA substitution.. Bioinformatics.

[89] Zwickl DJ (2006).

[90] Swofford DL (1998). PAUP*4.0: phylogenetic analysis using parsimony.

[91] Felsenstein J (2005). PHYLIP version 3.6.. http://evolution.gs.washington.edu/phylip.html.

[92] Ronquist F, Huelsenbeck JP (2003). MrBayes 3: Bayesian phylogenetic inference under mixed models.. Bioinformatics.

[93] Rambaut A, Drummond AJ (2009). Tracer version 1.5.. http://tree.bio.ed.ac.uk/software/tracer/.

[94] Drummond A, Rambaut A (2007). BEAST: Bayesian evolutionary analysis by sampling trees.. BMC Evolutionary Biology.

[95] Rambaut A, Drummond AJ (2010). TreeAnnotator version 1.6.. http://beast.bio.ed.ac.uk/TreeAnnotator.

[96] Moncalvo J-M, Wang H-H, Hseu R-S (1995). Phylogenetic relationships in *Ganoderma* inferred from the internal transcribed spacers and 25S ribosomal DNA sequences.. Mycologia.

[97] Riethmuller A, Voglmayr H, Goker M, Weiss M, Oberwinkler F (2002). Phylogenetic relationships of the downy mildews (Peronosporales) and related groups based on nuclear large subunit ribosomal DNA sequences.. Mycologia.

[98] White T, Bruns T, Lee S, Taylor J, Innis M, Gelfand D, Sninsky J, White T (1990). Amplification and direct sequencing of fungal ribosomal RNA genes for phylogenetics.. PCR Protocols: A Guide to Methods and Applications.

[99] Martin FN (2000). Phylogenetic relationships among some *Pythium* species inferred from sequence analysis of the mitochondrially encoded cytochrome oxidase II gene.. Mycologia.

[100] Martin FN (2008). Mitochondrial haplotype determination in the oomycete plant pathogen *Phytophthora ramorum*.. Current Genetics.

[101] Martin FN, Tooley PW, Blomquist C (2004). Molecular detection of *Phytophthora ramorum*, the causal agent of sudden oak death in California, and two additional species commonly recovered from diseased plant material.. Phytopathology.

