# Supplementary material for: Species Tree Estimation for the Late Blight Pathogen, Phytophthora infestans, and Close Relatives
Source: PLoS One. 2012 May 17;7(5):e37003. doi: 10.1371/journal.pone.0037003 (PMC3355167; doi:10.1371/journal.pone.0037003)
Supplement: Table S2 — Tests of between-subjects effects, weighted least squares regression (weighted by locus length). (DOC) [file pone.0037003.s002.doc]

Table S2. Tests of between-subjects effects, weighted least squares regression (weighted by locus length).

|  | | | | | |
| --- | --- | --- | --- | --- | --- |
|  | | | | | |
| Source | Type III Sum of Squares | df | Mean Square | F | Sig. |
| Corrected Model | 727.094^a^ | 74 | 9.826 | 17.029 | 0.000 |
| Intercept | 223.992 | 1 | 223.992 | 388.207 | 0.000 |
| Species | 231.113 | 4 | 57.778 | 100.137 | 0.000 |
| Locus | 47.765 | 14 | 3.412 | 5.913 | 0.000 |
| Species * Locus | 193.283 | 56 | 3.451 | 5.982 | 0.000 |
| Error | 323.693 | 561 | 0.577 |  |  |
| Total | 1739.329 | 636 |  |  |  |
| Corrected Total | 1050.787 | 635 |  |  |  |
| a) R^2^ = 0.692 (Adjusted R^2^ = 0.651). | | | | | |
|
