# Supplementary material for: Species Tree Estimation for the Late Blight Pathogen, Phytophthora infestans, and Close Relatives
Source: PLoS One. 2012 May 17;7(5):e37003. doi: 10.1371/journal.pone.0037003 (PMC3355167; doi:10.1371/journal.pone.0037003)
Supplement: Table S4 — Alternative maximum likelihood topologies for nuclear and mitochondrial datasets. (DOC) [file pone.0037003.s004.doc]

Table S4. Alternative maximum likelihood topologies for nuclear and mitochondrial datasets.

| STEM Topology | logL |
| --- | --- |
| *Nuclear* |  |
| ((((infestans, andinaA), (ipomoeae, andinaB)), mirabilis), phaseoli) | 121.71 |
| (((infestans, andinaA), (ipomoeae, andinaB), mirabilis), phaseoli) | 121.40 |
| ((((infestans, andinaA), ipomoeae, andinaB), mirabilis), phaseoli) | 121.20 |
| (((infestans, andinaA), (ipomoeae, andinaB, mirabilis)), phaseoli) | 120.98 |
| (((infestans, andinaA), (ipomoeae, andinaB)), mirabilis, phaseoli) | 120.86 |
| *Mitochondrial* |  |
| (((((infestans, andinaA), andinaB), ipomoeae), mirabilis), phaseoli) | -3417.30 |
| ((((infestans, andinaA), ipomoeae, andinaB), mirabilis), phaseoli) | -3421.49 |
| ((((infestans, andinaA), andinaB), ipomoeae, mirabilis), phaseoli) | -3426.45 |
| ((((infestans, andinaA, andinaB), ipomoeae), mirabilis), phaseoli) | -3427.97 |
| ((((infestans, andinaA), andinaB, mirabilis), ipomoeae), phaseoli) | -3430.64 |
| ((((infestans, andinaA, ipomoeae), andinaB), mirabilis), phaseoli) | -3431.90 |
| (((infestans, andinaA, andinaB), ipomoeae, mirabilis), phaseoli) | -3437.16 |
