# Supplementary material for: Species Tree Estimation for the Late Blight Pathogen, Phytophthora infestans, and Close Relatives
Source: PLoS One. 2012 May 17;7(5):e37003. doi: 10.1371/journal.pone.0037003 (PMC3355167; doi:10.1371/journal.pone.0037003)
Supplement: Table S5 — Minor splits and concordance factors found in the Bayesian Concordance Analysis. (DOC) [file pone.0037003.s005.doc]

Table S5. Minor splits and concordance factors found in the Bayesian Concordance Analysis.

| Split | CF | 95% CI |
| --- | --- | --- |
| *Nuclear* |  |  |
| (infestans, andinaA, andinaB, mirabilis) | (ipomoeae, phaseoli) | 0.170 | 0, 0.375 |
| (infestans, andinaA, mirabilis) | (andinaB, ipomoeae, phaseoli) | 0.158 | 0, 0.375 |
| (ipomoeae, mirabilis) | (infestans, andinaA, andinaB, phaseoli) | 0.156 | 0, 0.375 |
| (mirabilis, andinaB) | (infestans, andinaA, ipomoeae, phaseoli) | 0.153 | 0, 0.375 |
| (infestans, andinaA, andinaB) | (ipomoeae, mirabilis, phaseoli) | 0.140 | 0, 0.375 |
| (infestans, andinaA, ipomoeae) | (andinaB, mirabilis, phaseoli) | 0.119 | 0, 0.375 |
| (infestans, andinaA, ipomoeae, mirabilis) | (andinaB, phaseoli) | 0.116 | 0, 0.375 |
| *Mitochondrial* |  |  |
| (infestans, andinaA, ipomoeae) | (andinaB, mirabilis, phaseoli) | 0.273 | 0, 0.75 |
| (infestans, andinaA, mirabilis) | (andinaB, ipomoeae, phaseoli) | 0.214 | 0, 0.75 |
| (infestans, andinaB, ipomoeae) | (andinaA, mirabilis, phaseoli) | 0.092 | 0, 0.25 |
| (andinaA, andinaB, ipomoeae) | (infestans, mirabilis, phaseoli) | 0.091 | 0, 0.25 |
| (infestans, andinaA, andinaB) | (ipomoeae, mirabilis, phaseoli) | 0.062 | 0, 0.25 |
| (ipomoeae, mirabilis) | (infestans, andinaA, andinaB, phaseoli) | 0.057 | 0, 0.25 |
| (infestans, andinaA, ipomoeae, mirabilis) | (andinaB, phaseoli) | 0.057 | 0, 0.25 |

CF – concordance factor

CI – confidence interval
